# Assessing potentially inappropriate medication use among older adults in Central and Eastern Europe

**DOI:** 10.1080/07853890.2025.2579794

**Published:** 2025-11-04

**Authors:** Jovana Brkic, Jindra Reissigova, Betul Okuyan, Maja Ortner Hadziabdic, Valentina Marinkovic, Annemie Somers, Graziano Onder, Sofija Sesto, Oznur Altiparmak, Ingrid Kummer, Margita Drzaic, Daniela Fialova

**Affiliations:** a Department of Social and Clinical Pharmacy, Faculty of Pharmacy in Hradec Kralove, Charles University, Hradec Kralove, Czechia; b Department of Social Pharmacy and Pharmaceutical Legislation, Faculty of Pharmacy, University of Belgrade, Belgrade, Serbia; c Department of Statistical Modelling, Institute of Computer Science of the Czech Academy of Sciences, Prague, Czechia; d Department of Clinical Pharmacy, Faculty of Pharmacy, Marmara University, Istanbul, Turkey; e Centre for Applied Pharmacy, Faculty of Pharmacy and Biochemistry, University of Zagreb, Zagreb, Croatia; f Pharmaceutical Care Unit, Faculty of Pharmaceutical Sciences, Ghent University, Ghent, Belgium; g Department of Pharmacy, Ghent University Hospital, Ghent, Belgium; h Ageing, Neurosciences, Head-Neck and Orthopaedics Sciences, Fondazione Policlinico Universitario Agostino Gemelli IRCCS, Rome, Italy; i Department of Geriatrics and Orthopaedic Sciences, Universita Cattolica del Sacro Cuore, Rome, Italy; j Department of Clinical Pharmacy, Faculty of Pharmacy, Ege University, Izmir, Turkey; k City Pharmacies Zagreb, Zagreb, Croatia; l Department of Geriatrics and Gerontology, First Faculty of Medicine, Charles University, Prague, Czechia

**Keywords:** Potentially inappropriate medication list, aged, prevalence

## Abstract

**Objective:**

The aim of this study was to examine the prevalence of potentially inappropriate medication (PIM) use and its associated risk factors in community-dwelling older adults from five Central and Eastern European (CEE) countries.

**Materials and methods:**

This secondary analysis of a cross-sectional survey, which was part of the Horizon 2020 EuroAgeism ESR7 project, was conducted between February 2019 and March 2020 in Bulgaria, Croatia, Czechia, Estonia, and Serbia. We enrolled older adults aged ≥65 years who visited community pharmacies to acquire medications. The prevalence of PIM use was determined by applying all 282 criteria from the EU(7)-PIM list. Risk and protective factors for PIM use were evaluated using multiple logistic regression. R software version 4.3.2 was used in statistical analysis.

**Results:**

Most of the 2,155 participants were women (63.3%) and aged 65–74 years (64.8%). The overall PIM prevalence was 56.0% (95% confidence interval 53.8%–58.1%), ranging from 29.5% in Czechia to 70.0% in Croatia. The most commonly used PIMs were benzodiazepines (16.7% of all PIMs), followed by nonsteroidal anti-inflammatory drugs (14.3%), and proton pump inhibitors taken for more than 8 weeks (14.1%). Multiple logistic regression revealed that residence, increasing comorbidity burden, and polypharmacy were significant risk factors associated with PIM use in older adults.

**Conclusions:**

Our findings demonstrate a high prevalence of PIM use among older patients from CEE countries and considerable cross-country differences, underscoring the need to improve medication prescribing for older adults to improve healthcare quality and patient outcomes.

## KEY MESSAGES

- Potentially inappropriate medication use is widespread in Central and Eastern Europe.
- It is necessary to improve the prescribing process for older adults worldwide.

## Introduction

Older adults are more susceptible to adverse drug outcomes because physiological changes related to aging diminish the body’s compensatory mechanisms and alter its interactions with medications, known as pharmacokinetics and pharmacodynamics of drugs [1]. Additionally, older adults tend to have more health issues and disabilities, and to be frailer than younger adults; therefore, they are more likely to take more medications and experience medication-related harm [2]. This harm can occur due to the use of potentially inappropriate medications (PIMs), i.e. ineffective medications, medications that have more risks than benefits (especially when safer therapeutic alternatives exist) or medications prescribed without a clinical indication or at wrong doses, frequency, or duration of treatment [3].

PIM use is a global public health issue owing to its high prevalence across countries and adverse effects on older adults’ lives [4]. However, although recognized as a serious problem, it remains unclear whether interventions aimed to improve the issue have led to clinically significant improvements [5]. Recent systematic reviews have reported a global PIM prevalence of 33.3% in primary care settings [6] and 43.2% in long-term care (LTC) settings [7]. PIM use in older adults is associated with increased morbidity, increased healthcare service use, higher health care costs, and reduced quality of life [8–10]. This burden is especially high in frail older adults with increased vulnerability to acute stressors resulting from aging and disease-associated decline in reserve and function across multiple physiological systems, leading to an increased risk of adverse health outcomes [11,12]. Moreover, the negative clinical, economic, and humanistic consequences of PIM use can be more severe among older adults from less-developed and less-resourced regions and countries, such as Central and Eastern Europe (CEE) [13,14].

PIM use is assessed by explicit or implicit tools [15]. Implicit tools, such as the Medication Appropriateness Index [16], are based on a clinician’s or clinical pharmacist’s judgment of medication appropriateness for the individual patient [15]. Therefore, they depend on clinical knowledge and critical reasoning and are time-consuming to apply [17]. Conversely, more easily applicable explicit tools, such as the American Geriatrics Society Beers Criteria [18], STOPP/START version 3 criteria [19] and EU(7)-PIM list [20] are lists of medications/medication classes that should be avoided in older adults [15]. However, explicit tools do not consider all patient factors, such as comorbidities, disease progression, previous attempts to discontinue a PIM, and patient preference [21].

The issue of PIM use is understudied in the CEE region, as revealed in a systematic review [14]. Furthermore, only a few studies have examined the prevalence of PIM across countries: (1) in community dwellers Fialová et al. [22], Pineda et al. [23], and a pilot study by Tuula et al. [24]; (2) in outpatients Saka et al. [25]; (3) in hospitalized patients Gallagher et al. [26] and Saka et al. [27]; and, (4) in LTC residents Malek Makan et al. [28]. Therefore, to address the research gap on the PIM prevalence across countries, specifically in CEE countries this study aimed to (1) compare the prevalence of PIM use in community-dwelling older patients from five CEE countries (Bulgaria, Croatia, Czechia, Estonia, and Serbia) using the explicit tool, the EU(7)-PIM list [20] and (2) identify characteristics of the population at risk of PIM use to define a possible target for interventions aimed at improving prescribing appropriateness.

## Materials and methods

### Study design

This multicenter cross-sectional study involved the secondary analysis of the subset of data collected in the Horizon 2020 EuroAgeism ESR7 project titled ‘Inappropriate Prescribing and Availability of Medication Management Services in Older Patients in Europe’ [29]. One of the goals of the Horizon 2020 EuroAgeism ESR7 project was to examine the prevalence and determinants of PIM use in older adults, which is presented in this paper. Additionally, the results from the literature reviews conducted within the NETPHARM project were used to summarize the Discussion section.

The study was reported in accordance with the STrengthening the Reporting of Observational studies in Epidemiology (STROBE) Checklist: cross-sectional studies [30].

### Study setting

Data were collected from February 2019 to March 2020 in community pharmacies located in cities and towns that were not adjacent to hospitals. Community pharmacies and pharmacy chains were selected from three different regions or cities in each country. However, in Estonia, pharmacies were selected from four regions to account for smaller population sizes. The inclusion of pharmacies from selected regions/cities in our study was based on their willingness to participate.

#### Background information on the CEE countries included in this study

CEE countries share a common history, being former communist countries with similar healthcare systems [31]. Following the fall of communist regimes, the transition to market economies, and the breakup of several countries, these nations have diverged in their development. They generally lag behind the other European Union (EU) countries [31]. Here, we will briefly discuss the five countries included in our study: Bulgaria, Croatia (formerly part of Yugoslavia), Czechia (formerly part of Czechoslovakia), Estonia (formerly part of the Union of Soviet Socialist Republics, USSR), and Serbia (formerly part of Yugoslavia).

All countries are EU members except for Serbia, an EU candidate country. According to the World Bank classification of economies, Bulgaria and Serbia are upper-middle-income economies, while Croatia, Czechia, and Estonia are high-income economies [32]. Key country indicators, namely, life expectancy at birth, life expectancy at age 65, gross domestic product (GDP) per capita, health expenditure per capita, and the share of GDP allocated to health, are all below the EU average (see Table A1, Appendix, supplementary material). Further information on the health systems and pharmaceutical care is provided in Appendix, supplementary material Tables A2 and A3.

**Table 1.** Characteristics of the study population^a^.

	Overall (*n* = 2,155)	Bulgaria (*n* = 543)	Croatia (*n* = 391)	Czechia (*n* = 450)	Estonia (*n* = 311)	Serbia (*n* = 460)	*p*-value
Sociodemographic characteristics							
Sex, women, f (%)	1,351 (63.3)	334 (63.9)	248 (63.4)	281 (62.4)	219 (70.4)	269 (58.5)	0.021
Age (years)							
Range	65–99	65–99	65–92	65–97	65–98	65–93	
Mean (SD)	73.0 (6.8)	72.5 (6.7)	74.3 (6.6)	71.7 (6.3)	73.7 (7.1)	73.1 (7.0)	<0.001
Category, f (%)							<0.001
65–74	1,389 (64.8)	365 (68.1)	217 (56.2)	330 (73.3)	185 (59.5)	292 (63.5)	
75–84	584 (27.3)	131 (24.4)	135 (35.0)	96 (21.3)	96 (30.9)	126 (27.4)	
≥85	170 (7.9)	40 (7.5)	34 (8.8)	24 (5.3)	30 (9.6)	42 (9.1)	
Marital status, married, f (%)	1,276 (59.3)	298 (55.1)	228 (58.5)	325 (72.2)	144 (46.3)	281 (61.1)	<0.001
Education, f (%)							<0.001
No schooling, primary school	490 (22.9)	91 (17.1)	99 (25.4)	110 (24.4)	47 (15.1)	143 (31.1)	
High School	1,037 (48.4)	230 (43.2)	186 (47.8)	258 (57.3)	156 (50.2)	207 (45.0)	
Bachelor’s, Master’s, or higher degree	616 (28.7)	212 (39.8)	104 (26.7)	82 (18.2)	108 (34.7)	110 (23.9)	
Health characteristics							
Diseases and conditions, median (IQR)	4 (2–5)	3 (2–5)	5 (3–7)	3 (2–5)	3 (2–5)	4 (2–5)	<0.001
Most prevalent diseases and conditions, f (%)							
Hypertension	1,566 (72.8)	371 (68.3)	293 (75.5)	311 (69.1)	228 (73.3)	363 (78.9)	0.001
Chronic pain	984 (45.8)	327 (60.8)	197 (50.6)	165 (36.7)	140 (45.0)	155 (33.7)	<0.001
Dyslipidemia	605 (28.1)	82 (15.1)	186 (47.9)	176 (39.1)	49 (15.8)	112 (24.3)	<0.001
Diabetes mellitus	523 (24.3)	134 (24.7)	98 (25.3)	100 (22.2)	71 (22.8)	120 (26.1)	0.653
Sleep disorders	383 (17.8)	26 (4.8)	130 (33.5)	79 (17.6)	36 (11.6)	112 (24.3)	<0.001
Self-perceived health, good, f (%)	977 (45.4)	218 (40.4)	197 (50.5)	288 (64.0)	97 (31.2)	177 (38.5)	<0.001
Medication use, median (IQR)	4 (2–6)	3 (2–5)	5 (3–7)	3 (2–5)	5 (2–7)	5 (3–6)	<0.001
Health care utilization							
Hospitalization in the previous year, f (%)	533 (25.3)	91 (17.7)	51 (13.8)	36 (8.0)	287 (92.3)	68 (14.8)	<0.001
Emergency department visit in the previous year, f (%)	361 (16.8)	78 (14.6)	97 (24.9)	13 (2.9)	105 (33.8)	68 (14.8)	<0.001
Physician visits in the previous year, median (IQR)	5 (3–10)	8 (4–14)	7 (4–12)	4 (3–6)	3 (2–5)	6 (4–13)	<0.001

Note: f, absolute frequency; IQR, interquartile range (first quartile–third quartile).

^a^ Calculated from nonmissing values; the number of missing values <5%, except for multimorbidity (8.8% in Bulgaria, 9.0% in Croatia), hospitalization in the previous year (5.2% in Bulgaria, 5.4% in Croatia), physician visits in the previous year (18.7% in Czechia). Physician visits represent the sum of general practitioner (GP) and specialist visits.

**Table 2.** Number of potentially inappropriate medications (PIMs).

	Overall (*n* = 2,155)	Bulgaria (*n* = 543)	Croatia (*n* = 391)	Czechia (*n* = 450)	Estonia (*n* = 311)	Serbia (*n* = 460)	*p*-value
At least one PIM, f (%)							<0.001
No	924 (42.9)	258 (47.5)	111 (28.4)	316 (70.2)	99 (31.8)	140 (30.4)	
Yes	1175 (54.5)	267 (49.2)	259 (66.2)	132 (29.3)	201 (64.6)	316 (68.7)	
NA	56 (2.6)	18 (3.3)	21 (5.4)	2 (0.4)	11 (3.5)	4 (0.9)	
Number of PIMs, f (%)							<0.001
0	924 (42.9)	258 (47.5)	111 (28.4)	316 (70.2)	99 (31.8)	140 (30.4)	
1	609 (28.3)	163 (30)	90 (23)	98 (21.8)	84 (27)	174 (37.8)	
2	276 (12.8)	71 (13.1)	54 (13.8)	18 (4)	51 (16.4)	82 (17.8)	
3	128 (5.9)	12 (2.2)	38 (9.7)	11 (2.4)	32 (10.3)	35 (7.6)	
≥4	48 (2.2)	7 (1.3)	9 (2.3)	4 (0.9)	20 (6.4)	8 (1.7)	
NA	170 (7.9)	32 (5.9)	89 (22.8)	3 (0.7)	25 (8)	21 (4.6)	
Number of PIMs per PIM user, median (IQR)	1 (1–2)	1 (1–2)	2 (1–2)	1 (1–1.5)	2 (1–3)	1 (1–2)	<0.001

Note: f, absolute frequency; NA, not applicable because the information necessary to determine appropriateness, such as dose, dosing regimen, or duration of treatment, was missing. The number of PIMs could not be calculated when the information about the PIM was NA. The median (IQR) was calculated excluding NA values. IQR interquartile range (first quartile–third quartile).

The unadjusted prevalence of at using least one PIM (%) was: in all countries 56.0%, 95% CI 53.8%–58.1%, Czechia 29.5%, 95% CI 25.3%–33.9%; Bulgaria 50.9%, 95% CI 46.5%–55.2%; Estonia 67.0%, 95% CI 61.4%–72.3%; Serbia 69.3%, 95% CI 64.8%–73.5%; and Croatia 70.0%, 95% CI 65.0%–74.6%. Prevalence was calculated by excluding NA values.

**Table 3.** Proportion of potentially inappropriate medications (PIMs)^a^.

PIMs, f (%)	Overall (*n* = 1,958)	Bulgaria (*n* = 390)	Croatia (*n* = 473)	Czechia (*n* = 184)	Estonia (*n* = 405)	Serbia (*n* = 506)
Benzodiazepines	327 (16.7)	14 (3.6)	129 (27.3)	10 (5.4)	27 (6.7)	147 (29.1)
Diazepam	108 (5.5)	1 (0.3)	60 (12.7)	1 (0.5)	16 (4.0)	30 (5.9)
Bromazepam	95 (4.9)	8 (2.1)	1 (0.2)	3 (1.6)	3 (0.7)	80 (15.8)
Alprazolam	87 (4.4)	5 (1.3)	53 (11.2)	5 (2.7)	8 (2.0)	16 (3.2)
Lorazepam (> 1 mg/day)	22 (1.1)	0 (0.0)	5 (1.1)	0 (0.0)	0 (0.0)	17 (3.4)
Nitrazepam	13 (0.7)	0 (0.0)	10 (2.1)	0 (0.0)	0 (0.0)	3 (0.6)
Chlordiazepoxide	1 (0.1)	0 (0.0)	0 (0.0)	1 (0.5)	0 (0.0)	0 (0.0)
Clobazam	1 (0.1)	0 (0.0)	0 (0.0)	0 (0.0)	0 (0.0)	1 (0.2)
Nonsteroidal anti-inflammatory drugs (NSAIDs)	280 (14.3)	46 (11.8)	63 (13.3)	21 (11.4)	75 (18.5)	75 (14.8)
Diclofenac	118 (6.0)	20 (5.1)	18 (3.8)	5 (2.7)	19 (4.7)	56 (11.1)
Ibuprofen (>3 × 400 mg/day or for a period longer than one week)	47 (2.4)	2 (0.5)	16 (3.4)	9 (4.9)	14 (3.5)	6 (1.2)
Ketoprofen	25 (1.3)	0 (0.0)	15 (3.2)	2 (1.1)	7 (1.7)	1 (0.2)
Meloxicam	23 (1.2)	4 (1.0)	3 (0.6)	2 (1.1)	11 (2.7)	3 (0.6)
Etoricoxib	18 (0.9)	4 (1.0)	1 (0.2)	0 (0.0)	12 (3.0)	1 (0.2)
Dexketoprofen	16 (0.8)	7 (1.8)	1 (0.2)	0 (0.0)	6 (1.5)	2 (0.4)
Aceclofenac	10 (0.5)	4 (1.0)	0 (0.0)	2 (1.1)	0 (0.0)	4 (0.8)
Piroxicam	9 (0.5)	4 (1.0)	3 (0.6)	0 (0.0)	2 (0.5)	0 (0.0)
Indometacin	6 (0.3)	1 (0.3)	3 (0.6)	1 (0.5)	1 (0.2)	0 (0.0)
Naproxen (>2 × 250 mg/day or for a period longer than one week)	4 (0.2)	0 (0.0)	2 (0.4)	0 (0.0)	0 (0.0)	2 (0.4)
Celecoxib	2 (0.1)	0 (0.0)	1 (0.2)	0 (0.0)	1 (0.2)	0 (0.0)
Lornoxicam	2 (0.1)	0 (0.0)	0 (0.0)	0 (0.0)	2 (0.5)	0 (0.0)
Proton pump inhibitors (PPIs) (>8 weeks)	277 (14.1)	16 (4.1)	76 (16.1)	42 (22.8)	82 (20.2)	61 (12.1)
Trimetazidine	75 (3.8)	7 (1.8)	15 (3.2)	1 (0.5)	9 (2.2)	43 (8.5)
Sulfonylureas	60 (3.1)	16 (4.1)	4 (0.8)	13 (7.1)	12 (3.0)	15 (3.0)
Glimepiride	59 (3.0)	16 (4.1)	3 (0.6)	13 (7.1)	12 (3.0)	15 (3.0)
Glibenclamide	1 (0.1)	0 (0.0)	1 (0.2)	0 (0.0)	0 (0.0)	0 (0.0)
Benzodiazepine-related drugs	57 (2.9)	2 (0.5)	22 (4.7)	7 (3.8)	22 (5.4)	4 (0.8)
Zolpidem (>5 mg/day)	38 (1.9)	0 (0.0)	22 (4.7)	7 (3.8)	5 (1.2)	4 (0.8)
Zopiclone (>3.75 mg/day)	19 (1.0)	2 (0.5)	0 (0.0)	0 (0.0)	17 (4.2)	0 (0.0)
Moxonidine	56 (2.9)	22 (5.6)	22 (4.7)	3 (1.6)	4 (1.0)	5 (1.0)

Note: f, absolute frequency.

^a^ The table presents the PIMs/PIM groups with an occurrence of ≥50; the rest of the PIMs are shown in Table A4 in the Appendix, supplementary material. Percentages were calculated as the proportion of all PIMs. A total of 183 medications were not included among the PIMs because it was not possible to determine whether they were PIMs (information such as dose, dosing regimen, or duration of treatment was missing).

### Participants

Older adults (≥65 years) who visited community pharmacies in five CEE countries (Bulgaria, Croatia, Czechia, Estonia, and Serbia) to acquire medications were included in our study. The following patients were excluded from the study: patients with severe cognitive impairment or severe communication problems (hearing or speech) who were unable to respond to research questions and provide informed consent, those with recent acute health worsening requiring hospitalization or emergency department visit, those with limited life expectancy (<1 year), or those receiving palliative or terminal care.

We used convenience sampling to collect data from consecutive community pharmacy patients who were willing to participate in the study. The refusal rate was <10% at all study sites.

### Sample size

The Horizon 2020 EuroAgeism ESR7 project aimed to estimate the prevalence of PIM use in each participating country. To achieve a 95% confidence interval for prevalence with a margin of error of 5%, the calculated required sample size per country was 385 patients. This calculation was based on the standard formula for estimating proportion: where corresponds to the 95% confidence level, was used as a conservative estimate of prevalence to maximize sample size, and represents the desired margin of error. However, in Estonia, the target sample size was not reached due to the relatively small total population size and, accordingly, to a smaller number of older adults, resulting in the recruitment of 311 older patients.

### Data collection

In all participating countries in the EuroAgeism H2020 ESR7 project, trained researchers, pharmacists, or pharmacy students interviewed patients who visited community pharmacies (not their family members or caregivers) using a structured questionnaire. Furthermore, when feasible, trained researchers complemented data gathered from patient interviews with information from pharmacy dispensing data (obtained from interviews with pharmacists), patient medical records (obtained from interviews with physicians or documentation provided by patients**—**patient reports, discharge letters, or patient information in the e-health system), and laboratory results (obtained from patient reports, discharge letters, or patient information in the e-health system). In all countries, pharmacists could only access prescription/dispensing information and did not have access to the medical records of patients. Only Estonian patients could access their e-health records online, and only Estonian health data were integrated from different healthcare providers. The questionnaire was piloted on ten older patients from Czechia, and the collected pilot data were not analysed in this study. Bilingual researchers translated it into local languages and back-translated it into English using the Brislin translation method. The questionnaire included items on sociodemographic characteristics, healthcare use, diseases, conditions, symptoms, functional status, and laboratory findings. In addition, comprehensive information about dietary supplements and medications, including as-needed medications and over-the-counter medications used in the past 7 days, was collected. This included brand and International Nonproprietary Names, assigned Anatomical Therapeutic Chemical (ATC) codes, drug strength, dosage form, dose, and dosage.

### Measurement tool

Our primary outcome**—**prevalence of PIM use**—**was determined by using the EU(7)-PIM list [20] with all items and without any adaptations. We decided to use the EU(7)-PIM list because: 1) it is an explicit tool relevant to the European region that was developed based on different regions in Europe, including Finland and Sweden in Scandinavia, France and Spain in southern Europe, Germany, and the Netherlands in central Europe, and Estonia in eastern Europe [20]; and, 2) the tool can be applied in a community pharmacy setting without any adaptations as it does not require comprehensive clinical information to define inappropriate medications.

To avoid assessor measurement errors when applying criteria to our extensive dataset, we developed algorithms in R software (version 4.3.2) (R Core Team, Vienna, Austria) for the assessment of PIMs. We also manually checked 41 of 282 (14.5%) algorithms that required additional information (such as dose, dosing regimen, or duration of treatment) in addition to a single ATC code.

### Data analyses

Data normality was assessed visually using a quantile-quantile plot (Q-Q plot) and the Shapiro–Wilk test. Normally distributed variables are summarized as mean and standard deviation (SD). Discrete variables are presented as median and interquartile range (IQR), and categorical variables are presented as absolute (f) and relative (%) frequencies.

Differences between categorical variables across countries were evaluated using the chi-square test or, in the case of at least one expected frequency of less than five, using Fisher’s exact test. Differences in mean age were tested using Welch’s one-way analysis of variance (ANOVA) (the unequal variances in normally distributed age were detected using the Levene test). The Kruskal–Wallis test was performed to compare discrete variables across countries.

The (unadjusted) prevalence of PIM use is expressed as the percentage of patients receiving at least one PIM. The 95% confidence interval (CI) was calculated using the Clopper–Pearson method.

We evaluated the potential predictors associated with PIM use using both univariable and multiple logistic regression analyses. We included previously reported potential predictors [33], including age, sex, country of residence, education, marital status, self-reported health, polypharmacy, comorbidity burden (sum of all identified chronic conditions out of 50 surveyed), hospitalization in the previous year, emergency department visits in the previous year, number of general practitioner (GP) visits, number of specialist visits, and total number of physician visits in the previous year (including the number of GP visits and the number of specialist visits). For continuous predictors, the assumption of linearity in the logistic regression was graphically checked (when the assumption was not met, the values were categorized based on graphical representation). The likelihood ratio test and Akaike information criterion (AIC, at least a 2-unit difference on AICs) were used to select significant predictors when performing the multiple logistic regression. In addition to the manual inclusion of selected predictors in the logistic regression model, a bidirectional stepwise selection procedure was also employed to identify the most significant predictors. A generalized version of the variance inflation factor (GVIF) was used to detect multicollinearity among the explanatory variables. Model fit was assessed using residual analysis and the Nagelkerke pseudo-R-squared coefficient. The discrimination ability of the logistic regression model was evaluated using the area under the receiver operating characteristic curve (c-statistic with the bootstrap 95% CI) and the calibration ability using the omnibus goodness of fit test. The logistic regression results are expressed as odds ratios (ORs) with corresponding Wald 95% CIs. The adjusted country-specific PIM prevalence was calculated using marginal probability prediction.

Missing values were not imputed, and an available case analysis was performed. A two-sided significance level (*p*-value) of <0.05 was considered statistically significant. Statistical analysis was performed using R software version 4.3.2 (R Core Team, Vienna, Austria).

### Ethical considerations

The study adhered to the Declaration of Helsinki. It was approved by the ethics committees of Medical University - Sofia, Bulgaria (099-120/15.01.2019), University of Zagreb, Faculty of Pharmacy and Biochemistry, Croatia (251-62-03-19-40), Charles University, Faculty of Pharmacy in Hradec Kralove, Czechia (0830/2019/B), University of Tartu, Estonia (298/T-2), and the University of Belgrade, Faculty of Pharmacy, Serbia (01-691/2). Written informed consent was obtained from all participants who were given the option to withdraw from the study at any time.

## Results

### Population characteristics

The main characteristics of the study population are summarized in Table 1. In total, 2,155 patients from five CEE countries were included in our study: 543 from Bulgaria, 391 from Croatia, 450 from Czechia, 311 from Estonia, and 460 from Serbia. Most of the patients were women (63.3%) and aged 65–74 years (64.8%). The median number (IQR) of diseases and conditions in older patients was 4 (2–5), with the most prevalent being hypertension (72.8%), chronic pain (45.8%), dyslipidemia (28.1%), diabetes mellitus (24.3%), and sleep disorders (17.8%). The median number (IQR) of medications per patient was 4 (2–6).

One-quarter of the patients (25.3%) had been hospitalized at least once in the preceding year. Furthermore, 16.8% of patients had visited the emergency department in the previous year.

### PIM use

The overall prevalence of PIM use considering all 282 EU(7)-PIM list [20] criteria was 56.0% (95% CI 53.8%–58.1%), ranging from 29.5% in Czechia to 70.0% in Croatia (Table 2). In total, 28.3% of the older adults were taking one PIM, 12.8% were taking two PIMs, and 8.2% were taking three or more PIMs. The number of PIMs could not be determined in 7.9% of the older adults.

The total number of PIMs detected was 1,958 (390 in Bulgaria, 473 in Croatia, 184 in Czechia, 405 in Estonia, and 506 in Serbia). We could not determine whether 183 medications were PIMs because information such as dose, dosing regimen, or duration of treatment was missing. The most prevalent PIMs are presented in Table 3, and the other less common ones are presented in Table A4 in the Appendix, supplementary material. We did not detect 171 of 282 (60.6%) PIMs listed in the EU(7)-PIM list [20]. The most common PIMs were benzodiazepines (327, 16.7% of all PIMs), followed by nonsteroidal anti-inflammatory drugs (NSAIDs) (280, 14.3%), and proton pump inhibitors (PPIs) taken for more than 8 weeks (277, 14.1%). The other EU(7)-PIM list [20] criteria that had a lower prevalence rate (ranked sixth) but are noteworthy are benzodiazepine-related drugs or ‘Z-drugs’ (57, 2.9%) because their adverse effect profiles are similar to those of benzodiazepines in older adults, which were the most prevalent PIMs.

**Table 4.** Multiple logistic regression results: predictors of potentially inappropriate medication (PIM) use.

Predictors (*n* = 2,017)	Odds ratio (OR)	95% confidence interval (CI)	*p*-value
Country			
Czechia	1.00		
Bulgaria	2.40	(1.80–3.20)	<0.001
Croatia	4.17	(2.99–5.81)	<0.001
Serbia	5.56	(4.10–7.55)	<0.001
Estonia	5.91	(4.20–8.33)	<0.001
Number of diseases and conditions			
0–1	1.00		
2–3	3.20	(2.32–4.42)	<0.001
4–5	5.71	(4.06–8.03)	<0.001
≥ 6	11.15	(7.65–16.25)	<0.001
Polypharmacy			
< 6	1.00		
≥ 6	1.57	(1.15–2.15)	0.005

Note: Polypharmacy (number of medications) does not include EU(7)-PIMs based on which groups of patients with and without potentially inappropriate medications (PIMs) were defined. Except for the predictors listed in Table 4, no additional predictors from Table A6, supplementary material, including sex and age, were statistically significant when tested simultaneously. Consequently, they were not included in the multiple logistic regression model, and the analysis was not adjusted for them.

The adjusted prevalence of using at least one PIM (%) in the individual countries is as follows: Czechia 30.5%, 95% CI 26.1%–35.3%; Bulgaria 51.3%, 95% CI 46.4%–56.1%; Croatia 64.6%, 95% CI 58.7%–70.2%; Serbia 70.9%, 95% CI 66.3%–75.1%; and Estonia 72.2%, 95% CI 66.6%–77.1%. Prevalence was adjusted for the number of diseases and conditions, and polypharmacy.

### Factors associated with PIM use

Table A5 in the Appendix, supplementary material summarizes the potential predictors of PIM use. Multiple logistic regression identified three factors that were significantly associated with PIM use when potential predictors were analyzed simultaneously. Patients living outside Czechia (OR for Bulgaria 2.40, 95% CI 1.80–3.20, OR for Croatia 4.17, 95% CI 2.99–5.81, OR for Serbia 5.56, 95% CI 4.10–7.55 and OR for Estonia 5.91, 95% CI 4.20–8.33), with a higher comorbidity burden (OR for 2–3 comorbidities 3.20, 95% CI 2.32–4.42, OR for 4–5 comorbidities 5.71, 95% CI 4.06–8.03 and OR for ≥6 comorbidities, 11.15, 95% CI 7.65–16.25) and with polypharmacy (OR 1.57, 95% CI 1.15–2.15), were at an increased odds of being prescribed a PIM (Table 4). The multiple logistic regression model had good properties (omnibus goodness of fit test *p* = 0.420; c-statistic 0.76, 95% CI 0.74–0.79; Nagelkerke pseudo-R-squared = 0.28). For each country, the prevalence of PIM adjusted for the number of diseases and conditions, and polypharmacy is shown below Table 4.

## Discussion

### Summary of the main results

Our study found a high prevalence of PIM use (56.0%, 95% CI 53.8–58.1%) in community-dwelling older adults from CEE, ranging from 29.5% in Czechia to 70.0% in Croatia.

The prevalence of PIM use is considerably lower in Czechia than in other countries. This lower prevalence could be because Czechia is a wealthier country with a considerably higher GDP per capita and considerably higher health expenditure per inhabitant than the other countries, complemented by high levels of accessibility and affordability of health care (Tables A1–A2, Appendix, supplementary material). Moreover, in Czechia, strategies to improve the appropriateness of medication use were introduced, such as physicians and pharmacists accessing a complete patient medication list (not containing other health data) to check polypharmacy, contraindications, and duplicate prescriptions. Furthermore, Czechia is the only country among the five CEE countries included in this study that has successfully implemented clinical pharmacy services in acute and ambulatory care and has started implementing them in LTC [34].

Studies have demonstrated that undergraduate and postgraduate education and training in geriatric medicine, as well as the density and number of geriatricians, vary widely across European countries [35–41]. Therefore, these differences in the breadth and depth of education and training in geriatrics, along with potential differences in education and training on appropriate prescribing for older adults and in awareness and knowledge about prescribing tools, might have influenced the difference in the prevalence of PIM across countries. Furthermore, the variability across countries could be due to differences in other socioeconomic and health system factors, culture, coordination and collaboration of health professionals, available medications on the market, their costs, reimbursement status, and copayment levels, prescribing limits, treatment guidelines, treatment traditions, prescribing practices, pharmaceutical promotion, and patient expectations.

### Agreements and disagreements with other studies or reviews

In this study, we observed a much higher overall PIM prevalence (56.0%) than that previously reported in two systematic reviews involving the general older population from CEE countries (34.6%) [14] and community-dwelling older adults from Europe (22.6%) [42]. However, it was considerably lower than the prevalence reported in a pilot study (96%) conducted in six CEE countries (Bulgaria, Hungary, Estonia, Latvia, Poland, and Romania) [24], but this pilot study included a small number of participants (*n* = 250) with polypharmacy (those who used five or more medications). It also used an assessment tool that was a combination of two instruments, the EU(7)-PIM list [20] and the FORTA (Fit fOR The Aged) List [43]. Conversely, our result was consistent with studies (a) that used the EU(7)-PIM list [20] in the community setting and reported PIM prevalences ranging from 22.6% to 83.7% [44–59], and (b) conducted in CEE countries (in all settings of care) that used the EU(7)-PIM list [20], and reported PIM prevalences ranging from 54.2% to 90.6% [48,50,51,60–65].

Whereas a few studies that have investigated PIM prevalence across countries revealed that it varied widely, 5.8%–41.1% [22] and 12.8%–49.4% [23] in community dwellers, 34.7%–77.3% and 22.7%–43.3% in hospitalized patients (determined by two different tools) [26], other studies showed similar prevalences, 84.4%–100% in community dwellers [24], 29.6%–35.2% in outpatients [25], 30.1%–32.1% in hospitalized patients [27], and 80.4%–93.3% in LTC residents [28]. Furthermore, systematic reviews have identified the variance of PIM prevalence across countries/regions: 1) in primary care—it was higher in the United Kingdom, Belgium, Australia, and New Zealand (35.9%–59.2%) than in the United States, Canada, the Netherlands, and middle-income countries (23.2%–29.9%) [6] and 2) in LTC—it was 49.0% (95% CI 42.5%–55.5%) in Europe, 26.8% (95% CI 16.5%–37.1%) in North America, and 29.8% (95% CI 19.3%–40.3%) in other countries [7].

The prevalence of PIM use in each country [Bulgaria (50.9%), Czechia (29.5%), Estonia (67.0%), and Serbia (69.3%)] was broadly in line with the findings of studies conducted in these countries in community settings: Bulgaria (67.0%–96.2%) [24,51], Czechia (15.7%–41.1%) [22], Estonia (97.9%) [24], and Serbia (27.3%–63.4%) [66–68]. No studies were conducted in a community setting in Croatia; however, the prevalence of PIM observed in our study (70.0%) was broadly in line with findings from other settings of care: acute 24.6%–68.8% [64,69,70] and outpatient 8.3%–62.4% [71,72]. Furthermore, when studies in these countries used the EU(7)-PIM list [20] as a tool, the prevalence of PIM use was 67.0% in Bulgaria (community setting) [51], 66.7% in Croatia (acute setting) [64], and 85.3% in Serbia (LTC setting) [65].

PIM prevalence depends on factors such as study context and participants. However, the PIM tool applied is the most important determinant of PIM prevalence, resulting in considerable differences between studies. These tools consider different medications inappropriate due to reasons such as diverse countries/regions of development with different medications available on their pharmaceutical markets and unique prescribing practices [73,74]. Moreover, many studies adapted PIM tools due to the lack of clinical information or differences in available medications on the market [42].

In this study, the most prevalent PIMs were benzodiazepines, followed by NSAIDs and PPIs administered for longer than 8 weeks. These results are consistent with systematic reviews assessing PIM use in older adults from Europe (community-dwelling) [42] and CEE (all settings of care) [14] that identified benzodiazepines as the most frequently used PIMs. Benzodiazepines have been identified among the top three most commonly used PIMs in all studies conducted in Croatia [64,69–72], Czechia [22,26,75,76], and Serbia [65–68,77,78]. Furthermore, when the EU(7)-PIM list [20] was used as a tool, the top PIMs were (1) benzodiazepines followed by PPIs in most studies from the CEE countries [48,50,51,60–65] and (2) PPIs followed by benzodiazepines and NSAIDs in most studies involving community-dwelling older adults [45–55,57–59]. Interestingly, a study conducted in Bulgaria [51] using the EU(7)-PIM list [20] found no prescribed benzodiazepines, PPIs, or NSAIDs.

The most commonly used PIMs identified in our study are significant causes of morbidity, mortality, hospitalization, and institutionalization in older adults. The prolonged use of PPI (>8 weeks) is associated with a number of adverse effects, such as *Clostridium difficile* infection, hypomagnesemia, and B12 deficiency [18,79,80]. An important negative outcome related to long-term PPI use in older adults is the increased risk of fractures [18,79,80]. Likewise, benzodiazepines cause fall-related fractures, among other adverse effects such as cognitive impairment, sedation, delirium, dependence, withdrawal syndrome, and psychomotor impairment [18,81,82]. In addition, older patients are at an increased risk of serious adverse effects of NSAID use, especially gastrointestinal bleeding, ulceration and perforation, and kidney injury [18,83,84,]. Therefore, it is recommended that NSAIDs be used at the lowest dose for the shortest period [18,83,84,].

Patients taking more medications were more likely to be prescribed a PIM, consistent with two systematic literature reviews that revealed a higher number of drugs to be the only consistent risk factor for PIM use in community-dwelling older adults [33,42]. Furthermore, our study identified country of residence as a significant factor for PIM use, which aligns with the results of two meta-analyses that demonstrated variation in the prevalence of PIM use in primary and outpatient settings across countries and regions [6,85,]. Finally, our study found that a higher comorbidity burden was associated with higher odds of PIM use. However, two systematic reviews revealed that this factor had a positive, negative, or no association with PIM use in studies conducted in community-dwelling older adults [33,42].

### Limitations

This study has some limitations. First, we used convenience sampling, not random probabilistic sampling; thus, our study sample was not representative of the target population, and our study suffers from selection bias. Second, we could not collect the essential sociodemographic characteristics of nonresponders and compare them to those of responders. Third, the cross-sectional design allowed us to determine the association between risk factors and outcomes, not causation. Fourth, the primary source of the data was self-reporting. Therefore, recall bias cannot be ruled out, which might have led to underestimation of the outcome and risk factors such as polypharmacy and multimorbidity. Fifth, we assessed only one aspect of prescribing quality. We did not assess underprescribing, interactions, or other processes involved in medication use. Finally, as our study included healthier community-dwelling older adults from several countries, our results cannot be generalized outside these contexts.

## Conclusions

This study revealed a high prevalence of PIM use among older adults in CEE, highlighting considerable cross-country differences and associated risk factors. These findings underscore the need for international prescribing quality indicators to enhance patient safety and healthcare quality for older adults. Thus, the unique contributions of this study are filling the research gap on PIM prevalence in the under-researched CEE region and providing cross-country comparisons. However, limitations, such as the use of convenience sampling and cross-sectional design, must be considered when interpreting the results. Future research should focus on cross-national studies in different healthcare settings, longitudinal studies to establish causality, and qualitative analyses to understand the issue better. Researchers are encouraged to explore the impact of targeted interventions and policies to reduce PIM use and improve patient outcomes, and develop standardized prescribing guidelines. and an internationally comparable PIM tool that could be used seamlessly around the globe.

## Supplementary Material

Supplemental Material

## Data Availability

The data supporting this study’s findings are available from the corresponding author, JB, upon reasonable request.
