# Supplementary material for: Assessing potentially inappropriate medication use among older adults in Central and Eastern Europe
Source: Ann Med. 2025 Nov 4;57(1):2579794. doi: 10.1080/07853890.2025.2579794 (PMC12587801; doi:10.1080/07853890.2025.2579794)
Supplement: Supplemental Material [file IANN_A_2579794_SM8601.docx]

Table A1. Key country characteristics 2023 (or nearest year)

| **Country** | **Population** | **Proportion of population aged 65 and older (%)** | **Life expectancy at birth (years)** | **Life expectancy at age 65 (years)** | **GDP per capita in PPS** | **CHE in PPS per inhabitant** | **CHE as percentage of GDP (%)** | **OOP expenditure as percentage of CHE (%)** |
| --- | --- | --- | --- | --- | --- | --- | --- | --- |
| Bulgaria | 6,447,710 | 23.5 | 75.8 | 15.4 | 64 | 1,707.02 | 8.56 | 33.97 |
| Croatia | 3,850,894 | 22.7 | 78.6 | 17.1 | 76 | 1,785.71 | 8.07 | 9.40 |
| Czechia | 10,827,529 | 20.4 | 80.0 | 18.0 | 91 | 2,991.73 | 9.49 | 12.73 |
| Estonia | 1,365,884 | 20.2 | 78.8 | 18.3 | 81 | 2,077.07 | 7.06 | 23.16 |
| Serbia | 6,641,197 | 22.1 | 75.2 | 15.4 | 44 | 1,466.43 | 10.01 | 35.76 |
| EU | 448,753,823 | 21.3 | 81.5 | 19.5 | 100 | 3,562.06 | 10.87 | 14.52 |

Note: CHE, Current health expenditure; GDP, gross domestic product; EU, European Union; OOP, Out-of-pocket; PPS, purchasing power standards

Source: Eurostat Database

Table A2. Health systems characteristics

| **Country** | **Organization** | **Financing** | **Accessibility** |
| --- | --- | --- | --- |
| **Bulgaria** | Centralized | - Compulsory health insurance financed mainly from payroll contributions and, to a lesser extent, from government contributions for certain population groups  - Single-payer system  - Voluntary health insurance plays a small role | Access to healthcare is a serious problem, with the highest level of catastrophic health expenditure in the EU due to:  - the insurance coverage gap, about 12% of the population is uninsured  - high OOP payments, the highest in the EU (driven mainly by costs for outpatient medications)  - quarterly referral quotas for diagnostic tests and specialist care (when GPs reach quarterly referral quotas, patients can choose to wait, pay service OOP, or circumvent GPs and go directly to hospital emergency departments)  - uneven distribution of healthcare facilities and healthcare professionals across the country (concentrated in urban areas)  - shortages of GPs (although the overall physician density is higher than the EU average) and nurses; additionally, healthcare professionals are aging and emigrating and are dissatisfied with working conditions  Furthermore, the health system’s performance is hampered by excessively hospital-centered care, with the highest hospital admission rate in the EU and a high number of hospital admissions for conditions that could be managed effectively in outpatient care and by underdeveloped and under-resourced primary care. |
| **Croatia** | Centralized | - Compulsory health insurance financed mainly from payroll contributions and, to a lesser extent, from government contributions for certain population groups  - Single-payer system  - Voluntary health insurance is taken out by a large part of the population, taking a larger share of health expenditure than in many EU countries | Healthcare services are generally accessible and affordable due to:  - the nearly universal population coverage (over 99% of the population) and a comprehensive benefits package  - low OOP payments; OOP spending on health as a share of final household consumption is the lowest in the EU (mainly on pharmaceuticals and dental care), with only about 14 % of the population being subject to user charges because of either not being covered by voluntary (complementary) health insurance or not being exempted from paying  - however, geographical barriers to healthcare exist in rural, poorly populated, and remote areas, especially on islands, where healthcare facilities, pharmacies, and healthcare workers are scarce, causing the highest level of unmet medical needs due to the distance in the EU |
| **Czechia** | Decentralized  (highly specialized care in certain fields is centralized) | - Compulsory health insurance financed mainly from payroll contributions and, to a lesser extent, from government contributions for certain population groups  - Multi-payer system; seven insurers are partially competing by offering different supplementary benefits, with the largest company insuring about half of the population  - Voluntary health insurance plays a small role | High level of accessibility and financial protection because of:  - virtually 100% coverage of the population with a broad and comprehensive benefits package including OTC pharmaceuticals if prescribed by a physician and good access to innovative pharmaceuticals  - almost all health services are free, and OOP payments are low existing only for pharmaceuticals and medical devices (the highest share of OOP payments), out-of-hours outpatient care, above-standard medical procedures, and dental care  - accessibility requirements – time and distance – are defined by the law  - patients have access to outpatient specialists directly without a referral and without paying a user fee |
| **Estonia** | Centralized | - Compulsory health insurance financed mainly from payroll contributions and, to a lesser extent, from government contributions for certain population groups  - Single-payer system  - Voluntary health insurance plays a small role | Access to health care remains a challenge and causes the highest reported level of unmet medical needs in the EU because of:  - long waiting times (the main cause of unmet medical needs)  - coverage – about 4% of the population is uninsured, and about 10% of the working-age population experience temporary gaps in coverage  - high OOP payments largely for dental care and medications  - acute and growing shortages of family physicians and psychiatrists in some regions, and nurses throughout the system; the numbers of physicians, nurses, and pharmacists are below the EU average as healthcare professionals are retiring and emigrating, and too few professionals are being trained |
| **Serbia** | Centralized  (recently recentralized by transferring ownership of healthcare buildings and equipment to the national level) | - Compulsory health insurance financed mainly from payroll contributions and, to a lesser extent, from government contributions for certain population groups  - Single-payer system  - Voluntary health insurance plays a small role | Although 98% of the population is covered by health insurance, there are several barriers to accessing healthcare:  - financial because of high co-payments (mainly driven by outpatient medications)  - long waiting times  - uneven distribution of healthcare professionals around the country and shortages of some specialties (e.g., anaesthesiologists); contradictory, there is an excess number of unemployed health professionals (mainly younger ones), which, combined with low salaries, creates an incentive for emigration |

Note: * EU, European Union; GP, general practitioner; OOP, out-of-pocket; OTC, over-the-counter

Source: Bulgaria: Country Health Profile 2023; Bulgaria: Health system review; Bulgaria: Health system summary; Croatia: Country Health Profile 2023; Croatia: Health system review; Croatia: Health system summary; Czechia: Country Health Profile 2023; Czechia: Health system review; Czechia: Health system summary, 2023; Estonia: Country Health Profile 2023; Estonia: Health system review; Estonia: Health system summary; Health systems in action: Serbia: 2022 edition; Serbia: Health system review.

Table A3. Pharmaceutical care

| **Country** | **Sale of medications outside of the pharmacies** | **Sale of medications via the Internet** | **Prescribing by other healthcare professionals (other than a physician) or dispensing by other healthcare professionals (other than a pharmacist or pharmacy technician)** | **Specificities** |
| --- | --- | --- | --- | --- |
| **Bulgaria** | Yes, OTC medications | Yes, OTC medications | Yes, some medications can be directly dispensed by physicians or dentists | - Some patients choose to buy the medication at full price instead of visiting a physician for a prescription because reimbursement for certain medications is lower or equal to the user fee for a visit to a physician.  - Some patients in Bulgaria cannot afford prescribed medicines due to high co-payments |
| **Croatia** | Yes, OTC medications | Yes, OTC medications | No | / |
| **Czechia** | Yes, OTC medications | Yes, OTC medications | No | - Patients have a right to be informed by their physicians and pharmacist if there are available medications with similar therapeutic effects but fully reimbursed or with lower co-payments  - Physicians and pharmacists can access a complete patient medication list (not containing other health data) to check on polypharmacy, contraindications, and duplicate prescriptions.  - Patients receive information on prescribed medications in paper form or via email, SMS, or application. |
| **Estonia** | No | Yes, both OTC and prescription-only medications with a valid e-prescription (except the anabolic steroids, narcotic, and psychotropic drugs) | Yes, nurses can prescribe certain medications mainly for chronic conditions; also, midwives can prescribe a limited list of medicines | - Physicians have to prescribe medications by the INN otherwise, they have to justify prescribing by the brand name. Pharmacists have to offer different generics, including the cheapest one, and advise patients on the prices.  - An advanced e-health system enables physicians to prevent harmful polypharmacy by having comprehensive patient health-related information, all data on healthcare visits, and the entire patient prescription history, along with a comprehensive database of drug interactions. |
| **Serbia** | No | No | No | Patients can acquire prescription medications without having a prescription in pharmacies, except for medicinal products containing narcotics or certain psychotropic substances that are issued with a special medical prescription and other psychotropic medications and antibiotics that are under stricter control. |

Note: INN, International Nonproprietary Name; OTC, over-the-counter

Source: Bulgaria: Health system review; Croatia: Health system review; Czechia: Health system review; Estonia: Health system review; Estonia: Health system summary; Home pharmacies in Serbia: An insight into self-medication practice; Law on Medicines and Medical Devices; Opinions and attitudes of Czech citizens on selected issues of drugs in society; Republic of Estonia Agency of Medicines; Serbia: Health system review.

Table A4. Proportion of potentially inappropriate medications (PIMs) with an occurrence of < 50*

| **PIMs, f (%)** | **Overall** (n=1,958) | **Bulgaria** (n=390) | **Croatia** (n=473) | **Czechia** (n=184) | **Estonia** (n=405) | **Serbia**  (n=506) |
| --- | --- | --- | --- | --- | --- | --- |
| Amiodarone | 45 (2.3) | 13 (3.3) | 7 (1.5) | 7 (3.8) | 8 (2.0) | 10 (2.0) |
| Propafenone | 45 (2.3) | 20 (5.1) | 6 (1.3) | 4 (2.2) | 5 (1.2) | 10 (2.0) |
| Acenocoumarol | 36 (1.8) | 21 (5.4) | 0 (0.0) | 0 (0.0) | 0 (0.0) | 15 (3.0) |
| Apixaban | 35 (1.8) | 12 (3.1) | 1 (0.2) | 1 (0.5) | 20 (4.9) | 1 (0.2) |
| Rivaroxaban | 34 (1.7) | 2 (0.5) | 8 (1.7) | 3 (1.6) | 21 (5.2) | 0 (0.0) |
| Ranitidine | 28 (1.4) | 11 (2.8) | 10 (2.1) | 0 (0.0) | 0 (0.0) | 7 (1.4) |
| Digoxin | 27 (1.4) | 12 (3.1) | 0 (0.0) | 1 (0.5) | 7 (1.7) | 7 (1.4) |
| Dabigatran | 26 (1.3) | 5 (1.3) | 6 (1.3) | 2 (1.1) | 6 (1.5) | 7 (1.4) |
| Piracetam | 26 (1.3) | 22 (5.6) | 0 (0.0) | 0 (0.0) | 4 (1.0) | 0 (0.0) |
| Spironolactone (> 25mg/d) | 25 (1.3) | 7 (1.8) | 4 (0.8) | 5 (2.7) | 5 (1.2) | 4 (0.8) |
| Theophylline | 25 (1.3) | 1 (0.3) | 6 (1.3) | 3 (1.6) | 3 (0.7) | 12 (2.4) |
| Rilmenidine | 23 (1.2) | 7 (1.8) | 0 (0.0) | 16 (8.7) | 0 (0.0) | 0 (0.0) |
| Verapamil | 23 (1.2) | 7 (1.8) | 2 (0.4) | 1 (0.5) | 5 (1.2) | 8 (1.6) |
| Nifedipine (sustained-release) | 21 (1.1) | 7 (1.8) | 1 (0.2) | 0 (0.0) | 4 (1.0) | 9 (1.8) |
| Clonazepam | 19 (1.0) | 8 (2.1) | 1 (0.2) | 0 (0.0) | 1 (0.2) | 9 (1.8) |
| Tramadol (non-sustained-release) | 19 (1.0) | 0 (0.0) | 4 (0.8) | 6 (3.3) | 7 (1.7) | 2 (0.4) |
| Aluminium-containing antacids | 18 (0.9) | 4 (1.0) | 5 (1.1) | 1 (0.5) | 6 (1.5) | 2 (0.4) |
| Pentoxifylline | 16 (0.8) | 6 (1.5) | 0 (0.0) | 0 (0.0) | 6 (1.5) | 4 (0.8) |
| Carbamazepine | 15 (0.8) | 5 (1.3) | 4 (0.8) | 1 (0.5) | 3 (0.7) | 2 (0.4) |
| Solifenacin | 15 (0.8) | 6 (1.5) | 3 (0.6) | 5 (2.7) | 1 (0.2) | 0 (0.0) |
| Doxazosin | 13 (0.7) | 1 (0.3) | 4 (0.8) | 3 (1.6) | 5 (1.2) | 0 (0.0) |
| Paroxetine | 13 (0.7) | 2 (0.5) | 1 (0.2) | 2 (1.1) | 4 (1.0) | 4 (0.8) |
| Metildigoxin | 12 (0.6) | 5 (1.3) | 7 (1.5) | 0 (0.0) | 0 (0.0) | 0 (0.0) |
| Nicergoline | 12 (0.6) | 11 (2.8) | 0 (0.0) | 0 (0.0) | 0 (0.0) | 1 (0.2) |
| Propranolol | 12 (0.6) | 3 (0.8) | 1 (0.2) | 0 (0.0) | 2 (0.5) | 6 (1.2) |
| Hydroxyzine | 11 (0.6) | 11 (2.8) | 0 (0.0) | 0 (0.0) | 0 (0.0) | 0 (0.0) |
| Amitriptyline | 10 (0.5) | 1 (0.3) | 0 (0.0) | 0 (0.0) | 6 (1.5) | 3 (0.6) |
| Famotidine | 9 (0.5) | 6 (1.5) | 2 (0.4) | 0 (0.0) | 0 (0.0) | 1 (0.2) |
| Insulin, sliding scale | 9 (0.5) | 3 (0.8) | 1 (0.2) | 2 (1.1) | 2 (0.5) | 1 (0.2) |
| Pramipexole | 9 (0.5) | 7 (1.8) | 1 (0.2) | 0 (0.0) | 1 (0.2) | 0 (0.0) |
| Triptanes | 9 (0.5) | 1 (0.3) | 3 (0.6) | 1 (0.5) | 4 (1.0) | 0 (0.0) |
| Ginkgo biloba | 8 (0.4) | 1 (0.3) | 0 (0.0) | 0 (0.0) | 0 (0.0) | 7 (1.4) |
| Mebeverine | 8 (0.4) | 2 (0.5) | 2 (0.4) | 0 (0.0) | 3 (0.7) | 1 (0.2) |
| Tizanidine | 8 (0.4) | 0 (0.0) | 0 (0.0) | 0 (0.0) | 5 (1.2) | 3 (0.6) |
| Ivabradine | 7 (0.4) | 5 (1.3) | 0 (0.0) | 1 (0.5) | 0 (0.0) | 1 (0.2) |
| Tramadol (sustained-release) | 7 (0.4) | 0 (0.0) | 1 (0.2) | 4 (2.2) | 1 (0.2) | 1 (0.2) |
| Diltiazem | 6 (0.3) | 1 (0.3) | 1 (0.2) | 0 (0.0) | 2 (0.5) | 2 (0.4) |
| Iron supplements / Ferrous sulfate (>325 mg/d) | 6 (0.3) | 1 (0.3) | 2 (0.4) | 0 (0.0) | 2 (0.5) | 1 (0.2) |
| Loperamide (>2 days) | 6 (0.3) | 2 (0.5) | 2 (0.4) | 0 (0.0) | 1 (0.2) | 1 (0.2) |
| Pioglitazone | 6 (0.3) | 1 (0.3) | 1 (0.2) | 3 (1.6) | 0 (0.0) | 1 (0.2) |
| Sitagliptine | 6 (0.3) | 0 (0.0) | 4 (0.8) | 1 (0.5) | 1 (0.2) | 0 (0.0) |
| Sotalol | 6 (0.3) | 2 (0.5) | 3 (0.6) | 0 (0.0) | 1 (0.2) | 0 (0.0) |
| Trospium | 6 (0.3) | 0 (0.0) | 4 (0.8) | 0 (0.0) | 2 (0.5) | 0 (0.0) |
| Urapidil | 6 (0.3) | 0 (0.0) | 4 (0.8) | 2 (1.1) | 0 (0.0) | 0 (0.0) |
| Colchicin | 5 (0.3) | 5 (1.3) | 0 (0.0) | 0 (0.0) | 0 (0.0) | 0 (0.0) |
| Metoclopramide | 5 (0.3) | 0 (0.0) | 4 (0.8) | 0 (0.0) | 1 (0.2) | 0 (0.0) |
| Naftidrofuryl | 5 (0.3) | 0 (0.0) | 0 (0.0) | 0 (0.0) | 5 (1.2) | 0 (0.0) |
| Nitrofurantoin (>1 week) | 5 (0.3) | 0 (0.0) | 3 (0.6) | 0 (0.0) | 2 (0.5) | 0 (0.0) |
| Venlafaxine | 5 (0.3) | 0 (0.0) | 0 (0.0) | 4 (2.2) | 1 (0.2) | 0 (0.0) |
| Vildagliptine | 5 (0.3) | 0 (0.0) | 5 (1.1) | 0 (0.0) | 0 (0.0) | 0 (0.0) |
| Acarbose | 4 (0.2) | 3 (0.8) | 1 (0.2) | 0 (0.0) | 0 (0.0) | 0 (0.0) |
| Acetylsalicylic acid (>325 mg) | 4 (0.2) | 0 (0.0) | 0 (0.0) | 3 (1.6) | 0 (0.0) | 1 (0.2) |
| Clonidine | 4 (0.2) | 4 (1.0) | 0 (0.0) | 0 (0.0) | 0 (0.0) | 0 (0.0) |
| Fluoxetine | 4 (0.2) | 0 (0.0) | 0 (0.0) | 0 (0.0) | 3 (0.7) | 1 (0.2) |
| Maprotiline | 4 (0.2) | 0 (0.0) | 3 (0.6) | 0 (0.0) | 0 (0.0) | 1 (0.2) |
| Oxybutynine (non-sustained-release) | 4 (0.2) | 0 (0.0) | 0 (0.0) | 1 (0.5) | 3 (0.7) | 0 (0.0) |
| Sodium picosulfate | 4 (0.2) | 2 (0.5) | 0 (0.0) | 1 (0.5) | 1 (0.2) | 0 (0.0) |
| Amantadine | 3 (0.2) | 1 (0.3) | 2 (0.4) | 0 (0.0) | 0 (0.0) | 0 (0.0) |
| Bisacodyl (>3 days) | 3 (0.2) | 0 (0.0) | 2 (0.4) | 0 (0.0) | 1 (0.2) | 0 (0.0) |
| Chlorpromazine | 3 (0.2) | 0 (0.0) | 0 (0.0) | 0 (0.0) | 0 (0.0) | 3 (0.6) |
| Darifenacin | 3 (0.2) | 0 (0.0) | 2 (0.4) | 1 (0.5) | 0 (0.0) | 0 (0.0) |
| Aripiprazole | 2 (0.1) | 0 (0.0) | 1 (0.2) | 0 (0.0) | 0 (0.0) | 1 (0.2) |
| Clomipramine | 2 (0.1) | 2 (0.5) | 0 (0.0) | 0 (0.0) | 0 (0.0) | 0 (0.0) |
| Flupentixole | 2 (0.1) | 2 (0.5) | 0 (0.0) | 0 (0.0) | 0 (0.0) | 0 (0.0) |
| Fluvoxamine | 2 (0.1) | 0 (0.0) | 1 (0.2) | 1 (0.5) | 0 (0.0) | 0 (0.0) |
| Ropinirole | 2 (0.1) | 0 (0.0) | 2 (0.4) | 0 (0.0) | 0 (0.0) | 0 (0.0) |
| Selegiline | 2 (0.1) | 2 (0.5) | 0 (0.0) | 0 (0.0) | 0 (0.0) | 0 (0.0) |
| Baclofen | 1 (0.1) | 0 (0.0) | 0 (0.0) | 0 (0.0) | 1 (0.2) | 0 (0.0) |
| Biperiden | 1 (0.1) | 1 (0.3) | 0 (0.0) | 0 (0.0) | 0 (0.0) | 0 (0.0) |
| Clozapine | 1 (0.1) | 0 (0.0) | 0 (0.0) | 0 (0.0) | 0 (0.0) | 1 (0.2) |
| Dimenhydrinate | 1 (0.1) | 1 (0.3) | 0 (0.0) | 0 (0.0) | 0 (0.0) | 0 (0.0) |
| Ebastine | 1 (0.1) | 0 (0.0) | 0 (0.0) | 0 (0.0) | 1 (0.2) | 0 (0.0) |
| Flecainide | 1 (0.1) | 1 (0.3) | 0 (0.0) | 0 (0.0) | 0 (0.0) | 0 (0.0) |
| Haloperidol (>2 mg single dose; >5mg/d) | 1 (0.1) | 0 (0.0) | 0 (0.0) | 0 (0.0) | 0 (0.0) | 1 (0.2) |
| Lithium | 1 (0.1) | 0 (0.0) | 1 (0.2) | 0 (0.0) | 0 (0.0) | 0 (0.0) |
| Oestrogen (oral) | 1 (0.1) | 0 (0.0) | 1 (0.2) | 0 (0.0) | 0 (0.0) | 0 (0.0) |
| Otilonium bromide | 1 (0.1) | 0 (0.0) | 1 (0.2) | 0 (0.0) | 0 (0.0) | 0 (0.0) |
| Phenobarbital | 1 (0.1) | 0 (0.0) | 0 (0.0) | 0 (0.0) | 0 (0.0) | 1 (0.2) |
| Pinaverium | 1 (0.1) | 0 (0.0) | 1 (0.2) | 0 (0.0) | 0 (0.0) | 0 (0.0) |
| Promethazine | 1 (0.1) | 0 (0.0) | 0 (0.0) | 1 (0.5) | 0 (0.0) | 0 (0.0) |
| Risperidone (>6 weeks) | 1 (0.1) | 0 (0.0) | 0 (0.0) | 0 (0.0) | 0 (0.0) | 1 (0.2) |
| Terazosin | 1 (0.1) | 0 (0.0) | 0 (0.0) | 0 (0.0) | 0 (0.0) | 1 (0.2) |
| Topiramate | 1 (0.1) | 0 (0.0) | 0 (0.0) | 0 (0.0) | 0 (0.0) | 1 (0.2) |
| Trihexyphenidyl | 1 (0.1) | 0 (0.0) | 0 (0.0) | 0 (0.0) | 1 (0.2) | 0 (0.0) |
| Viscous paraffin (=Liquid paraffin) | 1 (0.1) | 1 (0.3) | 0 (0.0) | 0 (0.0) | 0 (0.0) | 0 (0.0) |

Note: f, absolute frequency

* The percentages are calculated as a proportion of all PIMs.

Table A5. Univariable logistic regression results: predictors of potentially inappropriate medication (PIM) use*

| **Predictors** | **Overall** (n=2,099) | **Patients without**  **PIMs** (n=924) | **Patients with**  **PIMs** (n=1,175) | **Odds ratio**  **(OR)** | **95% confidence interval (CI)** | ***p-value*** |
| --- | --- | --- | --- | --- | --- | --- |
| Country, f (%) |  |  |  |  |  |  |
| Czechia | 448 (21.3) | 316 (34.2) | 132 (11.2) | 1.00 |  |  |
| Bulgaria | 525 (25.0) | 258 (27.9) | 267 (22.7) | 2.48 | (1.90-3.24) | <0.001 |
| Croatia | 370 (17.6) | 111 (12.0) | 259 (22.0) | 5.59 | (4.15-7.57) | <0.001 |
| Serbia | 456 (21.7) | 140 (15.2) | 316 (26.9) | 5.40 | (4.08-7.20) | <0.001 |
| Estonia | 300 (14.3) | 99 (10.7) | 201 (17.1) | 4.86 | (3.56-6.68) | <0.001 |
| Sex – female, f (%) | 1,315 (63.2) | 568 (61.8) | 747 (64.3) | 1.12 | (0.93-1.33) | 0.234 |
| Age category, f (%) |  |  |  |  |  |  |
| 65–74 | 1,354 (64.8) | 661 (71.8) | 693 (59.4) | 1.00 |  |  |
| 75–84 | 567 (27.2) | 199 (21.6) | 368 (31.5) | 1.76 | (1.44-2.16) | <0.001 |
| ≥85 | 167 (8.0) | 61 (6.6) | 106 (9.1) | 1.66 | (1.19-2.32) | 0.003 |
| Marital status – married, f (%) | 1,247 (59.5) | 581 (62.9) | 666 (56.8) | 0.77 | (0.65-0.92) | 0.004 |
| Education, f (%) |  |  |  |  |  |  |
| No schooling/Primary school | 480 (23.0) | 185 (20.1) | 295 (25.3) | 1.00 |  |  |
| High School | 1,007 (48.3) | 457 (49.6) | 550 (47.2) | 0.76 | (0.60-0.94) | 0.013 |
| Bachelor’s, Master’s, or higher degree | 600 (28.7) | 280 (30.4) | 320 (27.5) | 0.72 | (0.56-0.91) | 0.007 |
| Number of diseases and conditions, f (%) |  |  |  |  |  |  |
| 0–1 | 301 (14.9) | 231 (26.0) | 70 (6.2) | 1.00 |  | <0.001 |
| 2–3 | 678 (33.6) | 353 (39.7) | 325 (28.8) | 3.04 | (2.25-4.15) | <0.001 |
| 4–5 | 549 (27.2) | 201 (22.6) | 348 (30.9) | 5.71 | (4.17-7.90) | <0.001 |
| ≥ 6 | 489 (24.2) | 105 (11.8) | 384 (34.1) | 12.07 | (8.61-17.12) | <0.001 |
| Self-perceived health – good, f (%) | 945 (45.1) | 504 (54.7) | 441 (37.6) | 0.50 | (0.42-0.59) | <0.001 |
| Polypharmacy (≥ 6 medications) | 345 (16.4) | 78 (8.4) | 267 (22.7) | 3.19 | (2.45-4.20) | <0.001 |
| Hospitalization in the previous year, f (%) | 520 (25.4) | 181 (20.1) | 339 (29.5) | 1.66 | (1.35-2.05) | <0.001 |
| Emergency department visit in the previous year, f (%) | 357 (17.1) | 101 (11.0) | 256 (21.9) | 2.27 | (1.77-2.92) | <0.001 |
| GP visits in the previous year, f (%) |  |  |  |  |  |  |
| 0-1 | 401 (19.8) | 212 (24.0) | 189 (16.5) | 1.00 |  |  |
| 2-6 | 1137 (56.1)) | 501 (56.7) | 636 (55.6) | 1.42 | (1.13-1.79) | 0.002 |
| ≥ 7 | 488 (24.1) | 170 (19.3) | 318 (27.8) | 2.10 | (1.60-2.75) | <0.001 |
| Specialist visits in the previous year, f (%) |  |  |  |  |  |  |
| 0 | 538 (26.2) | 278 (31.1) | 260 (22.4) | 1.00 |  |  |
| 1-3 | 1185 (57.7) | 492 (55.1) | 693 (59.8) | 1.51 | (1.23-1.85) | <0.001 |
| ≥ 4 | 329 (16.0) | 123 (13.8) | 693 (17.8) | 1.79 | (1.36-2.37) | <0.001 |
| Physician visits in the previous year, f (%) |  |  |  |  |  |  |
| 0-2 | 405 (20.3) | 216 (25.0) | 189 (16.7) | 1.00 |  |  |
| 3-10 | 1118 (56.0) | 481 (55.7) | 637 (56.2) | 1.51 | (1.21-1.90) | <0.001 |
| ≥ 11 | 473 (23.7) | 166 (19.2) | 307 (27.1) | 2.11 | (1.61-2.78) | <0.001 |

Note: f, absolute frequency; GP, general practitioner; polypharmacy (number of medications) does not include PIMs based on which the groups of patients (with and without PIMs) were defined.

*Calculated from nonmissing values; the number of missing values <5%, except for physician visits in the previous year (6.6% in patients without a PIM). Physician visits represent the sum of general practitioner (GP) and specialist visits.

**References:**

*Database—Eurostat*. (n.d.). Retrieved May 17, 2024, from <https://ec.europa.eu/eurostat/data/database>

European Observatory on Health Systems and Policies, Bryndová, L., Šlegerová, L., Votápková, J., Hroboň, P., Shuftan, N., Horschig, M., & Spranger, A. (2023). *Czechia: Health system summary, 2023*. World Health Organization. Regional Office for Europe. <https://iris.who.int/handle/10665/374194>

European Observatory on Health Systems and Policies, Bryndová, L., Šlegerová, L., Votápková, J., Hroboň, P., Shuftan, N., & Spranger, A. (2023). *Czechia: Health system review*. World Health Organization. Regional Office for Europe. <https://apps.who.int/iris/handle/10665/366529>

European Observatory on Health Systems and Policies, Dimova, A., Rohova, M., Koeva, S., Atanasova, E., Koeva-Dimitrova, L., Kostadinova, T., Spranger, A., & Polin, K. (2022). *Bulgaria: Health system summary*. World Health Organization. Regional Office for Europe. <https://apps.who.int/iris/handle/10665/365286>

European Observatory on Health Systems and Policies, Džakula, A., Vočanec, D., Banadinović, M., Vajagić, M., Lončarek, K., Lovrenčić, I. L., Radin, D., & Rechel, B. (2021). *Croatia: Health system review*. World Health Organization. Regional Office for Europe. <https://iris.who.int/handle/10665/348070>

European Observatory on Health Systems and Policies, Džakula, A., Vočanec, D., Banadinović, M., Vajagić, M., Lončarek, K., Lovrenčić, I. L., Radin, D., & Rechel, B. (2022). *Croatia: Health system summary*. World Health Organization. Regional Office for Europe. <https://apps.who.int/iris/handle/10665/361153>

European Observatory on Health Systems and Policies, Habicht, T., Kahur, K., Kasekamp, K., Köhler, K., Reinap, M., Võrk, A., Sikkut, R., Aaben, L., Van Ginneken, E., & Webb, E. (2023). *Estonia: Health system summary, 2022*. World Health Organization. Regional Office for Europe. <https://apps.who.int/iris/handle/10665/366157>

European Observatory on Health Systems and Policies, Kasekamp, K., Habicht, T., Võrk, A., Köhler, K., Reinap, M., Kahur, K., Laarmann, H., & Litvinova, Y. (2023). *Estonia: Health system review*. World Health Organization. Regional Office for Europe. <https://iris.who.int/handle/10665/374315>

European Observatory on Health Systems and Policies & World Health Organization. Regional Office for Europe. (2022). *Health systems in action: Serbia: 2022 edition*. World Health Organization. Regional Office for Europe. <https://apps.who.int/iris/handle/10665/363482>

Kostriba, J., Kotlarova, J., & Vlcek, J. (2015). Opinions and attitudes of Czech citizens on selected issues of drugs in society. *Acta Poloniae Pharmaceutica—Drug Research,* *72*(5), 1027–1038.

Kusturica, M. P., Tomic, Z., Bukumiric, Z., Ninkovic, L., Tomas, A., Stilinovic, N., & Sabo, A. (2015). Home pharmacies in Serbia: An insight into self-medication practice. *International Journal of Clinical Pharmacy*, *37*(2), 373–378. <https://doi.org/10.1007/s11096-015-0071-x>

*Law on Medicines and Medical Devices [Zakon o lekovima i medicinskim sredstvima]. Official Gazette of the Republic of Serbia, 30/2010,107/2012, 113/2017 and 107/2017.* (2010). <https://www.pravno-informacioni-sistem.rs/SlGlasnikPortal/eli/rep/sgrs/skupstina/zakon/2010/30/7/reg>

OECD & European Observatory on Health Systems and Policies. (2023). *Bulgaria: Country Health Profile 2023*. *State of Health in the EU.* OECD Publishing, Paris. <https://doi.org/10.1787/8d90f882-en>

OECD & European Observatory on Health Systems and Policies. (2023). *Croatia: Country Health Profile 2023*. *State of Health in the EU.* OECD Publishing, Paris. <https://doi.org/10.1787/8a7eadc9-en>

OECD & European Observatory on Health Systems and Policies. (2023). *Czechia: Country Health Profile 2023*. *State of Health in the EU.* OECD Publishing, Paris. <https://doi.org/10.1787/24a9401e-en>

OECD & European Observatory on Health Systems and Policies. (2023). *Estonia: Country Health Profile 2023*. *State of Health in the EU.* OECD Publishing, Paris. <https://doi.org/10.1787/bc733713-en>

Republic of Estonia Agency of Medicines (n.d.). *Homepage*. Retrieved January 1, 2024, from <https://ravimiamet.ee/ravimite-kaitlemine/ravimi-kaitlemine/internetiapteek>

World Health Organization. Regional Office for Europe, European Observatory on Health Systems and Policies, Bjegovic-Mikanovic, V., Vasic, M., Vukovic, D., Jankovic, J., Jovic-Vranes, A., Santric-Milicevic, M., Terzic-Supic, Z., & Hernández-Quevedo, C. (2019). *Serbia: Health system review*. World Health Organization. Regional Office for Europe. <https://apps.who.int/iris/handle/10665/331644>

World Health Organization. Regional Office for Europe, European Observatory on Health Systems and Policies, Dimova, A., Rohova, M., Koeva, S., Atanasova, E., Koeva-Dimitrova, L., Kostadinova, T., & Spranger, A. (2018). *Bulgaria: Health system review*. World Health Organization. Regional Office for Europe. <https://apps.who.int/iris/handle/10665/330182>
